# Highlights from Heart Rhythm 2018: Novel Developments in the Field of Electrophysiology

**DOI:** 10.19102/icrm.2018.090910

**Published:** 2018-09-15

**Authors:** Claudio Tondo

**Affiliations:** ^1^Heart Rhythm Center at Monzino Cardiac Centre, IRCCS, Milan, Italy; ^2^Department of Clinical Sciences and Community Health University of Milan, Milan, Italy

**Keywords:** Atrial fibrillation, cardiac mapping, cardiac pacing, ventricular tachycardia

The annual congress of the Heart Rhythm Society (HRS) was held earlier this year in Boston, MA and, although some time has passed since, we are still echoing several comments about the anticipated clinical trials on the subject of catheter ablation of atrial fibrillation (AF) that were showcased and the new technologies in the fields of ablation and cardiac pacing that were presented. Therefore, in this short commentary on the latest developments in the field, I will spend some time on two of the major clinical trials of catheter ablation of AF and also dedicate special attention to the discussion of several novel technologies.

## Clinical trials on catheter ablation

Needless to say, the presentation of the results of the Catheter Ablation versus Antiarrhythmic Drug Therapy for AF (CABANA) clinical trial^1^ drew particular attention from both attendees and the industry at large. This multicenter trial enrolled more than 2,000 patients who were randomized to undergo either catheter ablation or drug therapy and had a composite primary endpoint of death, disabling stroke, major bleeding, and cardiac arrest. Notably, even though the use of catheter ablation prompted reductions of 33% and 40% in the primary endpoint and mortality, respectively, there were no significant differences in these parameters between the two groups on the basis of intention-to-treat analysis. The most likely reason for this finding is the high rate of crossover observed. However, the study did confirm that the higher rate of maintenance of sinus rhythm seen with ablation was also associated with a better quality of life.

A significant contribution to the understanding of the ominous relationship between AF and congestive heart failure comes from the impressive results of the Catheter Ablation versus Standard Conventional Treatment in Patients with Left Ventricular Dysfunction and AF (CASTLE-AF) clinical trial.^2^ Results of this randomized clinical trial showed that ablation for AF in patients with heart failure was associated with a significantly lower rate of a composite of death and hospitalization for heart failure as compared with medical treatment. Furthermore, a benefit was also demonstrated in all-cause mortality alone in the ablation group and in the significant reduction of the burden of AF with an improved ejection fraction. Therefore, this trial strongly corroborates the results of previous clinical studies conducted in smaller patient populations and may indicate that ablation in patients with heart failure is feasible, safe, and effective in the restoration of sinus rhythm.

## Clinical trials on catheter ablation

### Mapping

The electrophysiology community is still struggling with devising the most effective ablative approach to persistent and longstanding persistent AF. Bearing this in mind, different three-dimensional mapping systems (both invasive and noninvasive) have been investigated in the last few years. In this regard, it is worth mentioning again the AQ mapping system proposed by Acutus Medical (Carlsbad, CA, USA). The functioning of this system is based on the analysis of “dipole” density instead of “bipolar” voltage during mapping and has recently been improved in terms of signal interpretation, providing dipole density analysis of the electrical activity across the cell membrane with more specific details and thus favoring the detection of the core of the electrical process of the underlying complex atrial arrhythmia. The AQmap system (Acutus Medical, Carlsbad, CA, USA), based on the use of a 25-mm basket catheter with 48 ultrasound transducers, is capable of collecting up to 144,000 ultrasound points/minute and boasts 48 engineered electrodes for the collection of 150,000 intracardiac unipolar samples/second. The primary results^3^ of the use of the last version of the system appear to be very promising for the diagnosis and treatment of complex atrial arrhythmias.

### Pulmonary vein isolation

Balloon platforms for pulmonary vein (PV) isolation (PVI) in paroxysmal AF seem to be expanding, even in the field of radiofrequency (RF) ablation. Great interest has been shown in the data of a first-in-human clinical trial^4^ on the use of a multielectrode RF balloon in patients with paroxysmal AF. The trial, involving 39 patients, demonstrated a good level of maneuverability of the device for achieving PVI in 100% of participants, with acute reconnection occurring in 4.6% of them and with no major complications observed in any.

Another noteworthy study on the subject of PVI is an initial clinical report on the use of pulsed electric field (PEF) energy as an ultra-rapid, tissue-selective modality for cardiac ablation to obtain an effective PVI.^5^ This technology is based on the creation of microscopic pores in the cell membranes (electroporation), which is, by definition, a nonthermal ablation technique. The application of PEF energy is highly tissue-selective, as characteristic threshold field strengths favor necrosis in specific tissues. This technology appears to be very attractive for AF ablation, as cardiomyocytes have the lowest threshold values of any tissue and, therefore, PFE application allows for greater selectivity and can minimize the ablation of nontarget tissues (eg, esophagus, phrenic nerve). Furthermore, because of the biochemical process induced by the application of the PFE energy, there is no coagulative necrosis, thus reducing the risk of pulmonary stenosis. As presented during late-breaking clinical trials, the outcomes of PFE ablation were considered by Reddy et al.^5^ by encircling the PVs and posterior left atrium with a linear catheter and using a custom over-the-wire endocardial catheter for percutaneous transseptal PVI during concomitant cardiac surgery. A total of 22 patients were ablated, yielding a 100% PVI success rate with a procedure time of 67 minutes ± 10 minutes and a total PFE energy delivery time of less than 60 seconds/patient without complications. The data collected, although very preliminary, seem to pave a new avenue in the field of AF ablation with respect to the potential for an ultra-rapid, tissue-specific ablative approach.

### Ventricular tachycardia

Catheter ablation has already been deemed as an effective treatment for ventricular tachycardia (VT), but, despite the impressive improvements in efficacy achieved in the last few years, challenges regarding the procedure remain. This is mainly due to anatomical constraints that prevent the successful reaching of the arrhythmia substrate—for example, a VT of epicardial or intramural origin that cannot be easily reached with a conventional catheter from the endocardium. This has prompted investigators to devise different approaches to overcome some of the issues concerning the intramural substrate of VT. I think two of the latest approaches deserve to be mentioned. First, noninvasive stereotactic ablation has been recently tested in a few patients, and the two-year results on the feasibility of the technology were reported at HRS this year.^6^ The technique involves identifying the region of the heart containing the arrhythmia substrate and then irradiating that area, similar to tumor radiotherapy. This appears to be a revolutionary approach to arrhythmias, owing to its inherent noninvasive nature. Although promising, the main issue will be safety in the long-term, because of the potential for off-target (eg, the coronary arteries and other surrounding structures) exposure to radiation.

The second technique worth mentioning was presented as part of the late-breaking clinical trial sessions and involved the treatment of an intramural location of the circuit of the ventricular arrhythmia using a saline-irrigated needle to reach the deep substrate.^7^ The approach is based on the insertion of the needle into the myocardium, after which point, saline is delivered into the tissue via the needle and a radiofrequency current is applied across an electrode. The safety profile for this method has been tested in a small number of patients (31 patients) with nonischemic and ischemic VT requiring a median of 15 RF needle applications per patient, aiming at the RV/LV septum, and the initial findings are very encouraging. Currently, this approach is under investigation in a United States-based trial, and the investigators foresee its availability for general use within the next year or two.

### Heart failure

A promising perspective for patients with advanced (New York Heart Association functional classes III and IV) heart failure is the introduction of so-called cardiac contractility modulation (CCM), whose first clinical results from a randomized controlled trial (the FIX-HF-5C study) were presented^8^ as part of the late-breaking clinical trial sessions. This therapy consists of the delivery of nonexcitatory electrical signals to the heart during the absolute refractory period. The study was conducted in 160 patients with ejection fractions of between 25% and 45% and QRS durations < 130 ms who were randomized to conventional medical therapy (control group) or to continued medical therapy plus CCM (treatment group) for 24 weeks. As compared with the control group, the treatment group showed a better improvement of New York Heart Association functional class, six-minute hall walk score, and O_2_ consumption. Therefore, the trial met its prespecified objectives, confirming the safety and efficacy of CCM in patients with advanced congestive heart failure. These clinical results many thus represent a very promising adjunct therapy for this selected population of patients.

### Leadless pacing

Leadless pacemaker technology, initially introduced into clinical practice a few years ago, has already become a valuable resource in our pacing portfolio and has been implanted in more than 10,000 patients around the world. A significant contribution to the topic is represented in the long-term clinical results of the Micra™ Transcatheter Pacing System Post-Approval Registry.^9^ The registry included more than 1,700 patients, with an implantation success rate of 99.3% and a complication rate of 2.7% within 30 days after placement. It is worth mentioning that the risk of major complications (mainly pericardial effusion) with the Micra™ device (Medtronic, Minneapolis, MN, USA) was lower than that for patients with transvenous pacemakers and demonstrated biophysical parameters that remained stable through 12 months after implantation. Therefore, the long-term performance of the Micra™ transcatheter pacing system (Medtronic, Minneapolis, MN, USA) in international clinical practice remains consistent with the previously reported data,^10^ with infrequent major complications that occurred 64% less often as compared with in patients with conventional single-chamber transvenous pacemakers.
